# Overweight modifies the association between long-term ambient air pollution and prehypertension in Chinese adults: the 33 Communities Chinese Health Study

**DOI:** 10.1186/s12940-018-0401-2

**Published:** 2018-06-28

**Authors:** Bo-Yi Yang, Zhengmin Min Qian, Michael G. Vaughn, Steven W. Howard, John Phillip Pemberton, Huimin Ma, Duo-Hong Chen, Li-Wen Hu, Xiao-Wen Zeng, Chuan Zhang, Yan-Peng Tian, Min Nian, Xiang Xiao, Guang-Hui Dong

**Affiliations:** 1 Guangzhou Key Laboratory of Environmental Pollution and Health Risk Assessment; Guangdong Provincial Engineering Technology Research Center of Environmental and Health risk Assessment; Department of Preventive Medicine, School of Public Health, Sun Yat-sen University, Guangzhou, 510080 People’s Republic of China; 2 Department of Epidemiology, College for Public Health and Social Justice, Saint Louis University, Saint Louis, 63104 USA; 3 School of Social Work, College for Public Health and Social Justice, Saint Louis University, Saint Louis, 63104 USA; 4 Department of Health Management & Policy, College for Public Health & Social Justice, Saint Louis University, 3545 Lafayette Avenue, Saint Louis, MO 63104 USA; 5 State Key Laboratory of Organic Geochemistry and Guangdong Key Laboratory of Environmental Protection and Resources Utilization, Guangzhou Institute of Geochemistry, Chinese Academy of Sciences, Guangzhou, 510640 China; 6 Guangdong Environmental Monitoring Center, State Environmental Protection Key Laboratory of Regional Air Quality Monitoring, Guangdong Environmental Protection Key Laboratory of Atmospheric Secondary Pollution, Guangzhou, 510308 China

**Keywords:** Ambient air pollution, Chinese, Interaction, Overweight, Prehypertension

## Abstract

**Background:**

Research regarding the interaction of ambient air pollution and overweight on prehypertension is scarce. We aimed to test whether overweight modifies the association between long-term exposure to ambient air pollution and prehypertension in Chinese adults.

**Methods:**

A total of 16,188 Chinese adults, aged 18–74 years old, from 33 communities in 3 Northeastern Chinese cities were evaluated. Three-year average levels of particles with an aerodynamic diameter ≤ 10 μm (PM_10_), sulfur dioxides (SO_2_), nitrogen dioxides (NO_2_), and ozone (O_3_) were calculated at monitoring stations. Generalized additive models and 2-level regression analyses were applied.

**Results:**

We observed significant interactions between air pollutants and overweight on prehypertension and blood pressure. The associations of PM_10_, SO_2_, NO_2_, and O_3_ with prehypertension were significant among overweight participants (Prevalence Rate Ratios (PRRs) per interquartile range (IQR) of air pollutants: 1.14–1.20), but not among normal weight participants (PRRs: 0.98–1.04). PM_10_, SO_2_, and O_3_ were significantly associated with systolic blood pressure (SBP), and the magnitudes of these associations were higher among overweight adults (increases in SBP per IQR of air pollutants: 1.82–4.53 mmHg) than those among normal weight adults (increases in SBP: 0.42–0.61 mmHg). For diastolic blood pressure (DBP), significant associations were mainly observed in overweight participants (increases in DBP: 0.80–1.63 mmHg). Further stratified analyses showed that all these interactions were stronger in women, the older, and participants living in areas with lower income levels or higher population density.

**Conclusions:**

Being overweight may enhance the effects of ambient air pollution on prehypertension and blood pressure in Chinese adults.

**Electronic supplementary material:**

The online version of this article (10.1186/s12940-018-0401-2) contains supplementary material, which is available to authorized users.

## Background

Prehypertension, defined as blood pressure in the range of 120–139/80–89 mmHg, is a new category of blood pressure classification introduced by the Seventh Joint National Committee on the Prevention, Detection, Evaluation and Treatment of Hypertension (JNC-7) in 2003 [1]. Numerous studies have demonstrated that blood pressure in the prehypertension range was strongly related to increased risks of cardiovascular morbidity and mortality [2–4]. Prehypertension affects approximately 20–50% of adults worldwide, but its etiology is complex and poorly understood [4–6].

There is mounting evidence that exposure to short- and long-term ambient air pollution may raise blood pressure levels and result in a pro-hypertensive response [7–9]. Also, previous human and animal studies have indicated a strong relationship between overweight/obesity and higher blood pressure [10, 11]. The mechanisms by which ambient air pollutants could contribute to the development of prehypertension might include promoting systemic inflammation and oxidative stress, instigating autonomic dysfunction, and triggering vascular endothelial dysfunction [7]. The pathophysiological mechanisms of inflammation and oxidative stress are shared with overweight/obesity in the hypothesized etiology of prehypertension [12]. Laboratory evidence has suggested that overweight/obesity can facilitate the effects of inhaled ambient air pollution on adipose inflammation [13]. Thus, overweight/obese individuals may be more sensitive to the pro-hypertensive effects of ambient air pollutants.

Several human epidemiological studies have investigated the modification of obesity on the association between air pollution and health. A randomized-control study carried out among 348 participants in the United States revealed that exposure to fine particles < 2.5 μm in aerodynamic diameter (PM_2.5_) was associated with an increased risk of elevated pulse pressure among obese individuals [14]. Other large, population-based studies indicated that the effects of long-term exposure to particulate matter with an aerodynamic diameter ≤ 10 μm (PM_10_), sulfur dioxide (SO_2_), nitrogen dioxide (NO_2_), and ozone (O_3_) on hypertension were stronger among obese adults and children than those with normal body weight [15, 16]. Several studies have also investigated other health outcomes including coronary heart disease, stroke, QT interval (the time between the start of the Q wave and the end of the T wave in the heart’s electrical cycle), and heart rate variability, and obtained similar results [17–20]. However, to our knowledge, there is no published study that has specifically evaluated the effect of co-exposure to ambient air pollutants and overweight/obesity on prehypertension. One explanation for the lack of such an evaluation is that, although it is strongly associated with cardiovascular diseases and was defined by the JNC-7 over 10 years ago [1], prehypertension has yet to be widely adopted and given adequate attention [4, 5].

Given the current prehypertension and obesity epidemic, the ubiquitous nature of ambient air pollution, and the scarcity of such an evaluation, our main study objective was to fill this void in the research literature. Specifically, we tested the hypothesis that being overweight amplifies the effects of long-term exposure to ambient air pollutants on prehypertension and blood pressure in Chinese adults, using data from the 33 Communities Chinese Health Study (33CCHS).

## Methods

### Study city selection and subject recruitment

The 33CCHS was conducted in Liaoning province, which is situated in Northeast China and has a permanent population of over 20 million in 14 cities. In April 2009, to obtain the maximization pollution gradients, we selected three cities (Shenyang, Anshan, and Jinzhou) as study sites, according to the measured pollutant levels between 2006 and 2008.

The total number of districts from the three selected cities was 11, including five in Shenyang, three in Anshan, and three in Jinzhou. In each of the 11 districts, there was one available municipal air monitoring station. From communities within about 1-km of a monitoring station, we randomly selected 33 locales (each district had three locales). We randomly identified 700 to 1000 households within each locale. We then selected one participant, 18 to 74 years old from each household. Only participants who lived in the same household for more than five years were included. Finally, a total of 28,830 individuals were randomly selected, of whom 24,845 completed the survey and examination, resulting in an overall response rate of 86.2%. The analysis reported in the present study is restricted to 16,188 individuals after excluding participants who were already hypertensive. The study participants were younger, higher educated, excised more regularly, had higher incomes and body mass index (BMI), and had higher proportions of non-smoker, non-drinker and family history of hypertension, compared to those who were excluded from this study (hypertensive participants) (Additional file 1: Table S1). The study was approved by the Human Studies Committee of Sun Yat-Sen University. We collected written informed consent from all participants.

### Ambient air pollution data

Measurements of PM_10_, SO_2_, NO_2_, and O_3_ concentrations were obtained from the municipal air monitoring station in each district, using uniform criteria for monitoring, siting, instrumentation, and quality assurance to ascertain background air pollution concentrations. Continuous measurements of PM_10_, SO_2_, NO_2_, and O_3_ using β-attenuation, ultraviolet fluorescence, chemiluminescence, and ultraviolet photometry, respectively, were recorded by the district air monitoring stations. In order to assure that the monitoring stations were measuring the background air pollution, stations were required to be away from sources of emissions from fossil fuel or waste combustion such as major roadways and local industries. All measurements were required to meet standards established by the State Environment Protection Administration of China (1992) [21]. The continuous measures were used to generate 1-h concentration values that were then averaged into daily air pollution concentrations. Daily concentrations were defined as 24-h averages of PM_10_, SO_2_, and NO_2_ concentrations, and 8 h averages of O_3_ collected between the hours of 10:00 AM and 6:00 PM. The 3-year averages (2006–2008) were then calculated using the calculated daily averages, after excluding any days where at least 25% of the 1-h values were abnormal air pollution concentrations. The final exposure parameters therefore consisted of 3-year averages of daily pollution concentrations for PM_10_, SO_2_, NO_2_ and O_3_. Detailed air pollution data collection has been reported in our previous papers [15, 22] and in the Additional file 1: explanation of the air pollution data.

### Prehypertension

All investigators and staff in the 33CCHS study were required to successfully complete a training program according to the American Heart Association procedures [23]. Each trainee was required to take a qualification examination and was certified by the end of the training program. All participants were requested not to consume tea, coffee, alcohol or tobacco, and to abstain from exercising for over 30 min before blood pressure measurement. We measured systolic blood pressure (SBP) and diastolic blood pressure (DBP) three times after the participants had sat and rested for five minutes in a quiet and comfortable room. The standardized mercuric-column sphygmomanometer with an appropriate cuff size adapted to arm circumference was used to measure both SBP and DBP. The first reading was taken in both arms, and the second and third readings were taken on the arm showing higher blood pressure measurements. The average of three consecutive pairs of blood pressure measurements was recorded, with a 2-min interval between each measurement. According to the JNC-7 [1], prehypertension was defined as SBP of 120–139 mmHg or DBP of 80–89 mmHg and not taking antihypertensive medication. Normotension (including normotensive and hypotensive participants) was defined as having SBP of ≤120 mmHg and/or DBP of ≤80 mmHg and not receiving treatment for hypertension.

### Overweight

According to the protocols developed by the World Health Organization (WHO) [24], height was measured to the nearest 0.5 cm, with the participant’s back against a wall, no shoes, and eyes looking straight ahead, with a right-angle triangle placed on the top of the participant’s head and against the wall. Weight was measured to the nearest 0.1 kg with participants wearing no shoes and minimal outer garments. BMI was then calculated as weight in kilograms divided by the square of height in meters. According to the criteria proposed by the WHO, participants with a BMI < 25 kg/m^2^ were classified as normal weight (including participants with normal weight and underweight), a BMI of 25.0–29.9 kg/m^2^ were classified as overweight, and a BMI ≥30 kg/m^2^ were classified as obese. As the number of obese individuals was too small (*n* = 540) to deduce valid results, we combined overweight and obese individuals as a single group and labeled as “overweight”.

### Covariates

The following variables were included as covariates: age, sex (men vs. women), nationality (Han vs. others), household income (≤5000 Yuan, 5001–10,000 Yuan, 10,001–30,000 Yuan, ≥30,000 Yuan), education level achieved (no school, primary school, middle school, junior college or higher), smoking (smoker vs. non-smoker), alcohol consumption (consumer vs. non-consumer), exercise frequently (yes vs. no), controlled diet with low calorie and low fat (yes (occasionally, frequently, or everyday) vs. no (never or almost never)), sugar-sweetened soft drink consumption (≤ 1 day per week, 2–4 days per week, ≥5 days per week), family history of hypertension, per-capita gross domestic product (GDP) (an indicator of socioeconomic status (SES)) and population density (PD) in each district.

### Statistical analysis

Prior to proceeding with hypothesis testing, data normality and heterogeneity were assessed using the Shapiro-Wilks Test and the Bartlett test for unequal variances, respectively. Continuous variables were expressed as mean ± standard deviation (SD) and categorical variables as relative frequency percentages. Differences in the distribution of baseline characteristics between overweight and normal weight groups were tested using Student’s t-test for continuous variables and chi-square test for categorical variables. Scatter plots were used to explore the relationship between air pollutants and the prevalence of prehypertension (district-level data). In addition, age- and sex-adjusted prevalence rates of prehypertension were calculated according to categories of air pollutants concentrations (≥median value vs. <median value) and BMI (≥25 kg/m^2^ vs. < 25 kg/m^2^), as suggested by Turner and colleagues [25]. Generalized linear regression models were used to assess the association between ambient air pollutants and blood pressure. We applied a 2-level binary logistic regression model to examine the association between prehypertension and ambient air pollution (prevalence rate ratio (PRR) and corresponding 95% CI were calculated according to the method suggested by Schouten et al. [26]), using single-pollutant model. The participants were regarded as the first-level units and the districts as the second-level units, as described previously [22]. PM_10_, SO_2_, NO_2_ and O_3_ were classified as key exposure variables in the two-level logistic regression model. Analyses were adjusted using other key covariates (age, sex, race, education, income, smoking, drinking, exercise, diet, sugar-sweetened soft drink intake, family history of hypertension, GDP, and PD). In addition, we performed stratified analyses according to sex (men vs. women), age (< 60 years vs. ≥60 years), GDP level (low (< 73,459 Yuan) vs. high (≥73,459 Yuan)), and PD (low (< 8733 person/km^2^) vs. high (≥8733 person/km^2^)). An interaction term was added to the linear regression model to assess the significance of the effect modification. For the logistic regression model, we calculated the relative excess risk due to interaction (RERI) to assess the presence of interactions on the additive scale. An RERI of < 0, =0, and > 0 represents a negative interaction, no interaction, and positive interaction, respectively. We also performed sensitivity analyses by excluding participants who were underweight, hypotensive, or with diabetes mellitus. Furthermore, we examined the associations between air pollutants and blood pressures and prehypertension using multi-pollutant model. All analyses were conducted in SAS version 9.4 using the GLIMMIX procedure. The threshold for statistical significance was determined to be a 2-tailed *p*-value < 0.05.

## Results

The characteristics of the participants in this study are summarized in Table 1. Mean age of the 16,118 study participants was 42.31 years (SD = 12.75 years), 46.44% were males, 28.80% were smokers, 20.88% were alcohol consumers, and 31.74% had a family history of hypertension. The overall prevalence rates of prehypertension and overweight were 57.99 and 30.87%, respectively. Overweight participants differed from normal weight participants in being older, men, Han nationality, having higher household income, doing more regular exercise, consuming fewer sugar-sweetened beverages, and having higher prehypertension prevalence (All *p* < 0.05), but with similar levels of educational attainment, smoking and drinking status, practice of controlling diet with low calorie and low fat intake, and family history of hypertension.

**Table 1:** Characteristics of the study participants

	Normal weight^b^	Overweight^c^	Total
Characteristics	(*n* = 11,190)	(*n* = 4998)	(*n* = 16,188)
Age (years, mean ± SD)^a^	41.59 ± 12.96	43.90 ± 12.13	42.31 ± 12.75
Sex^a^			
Men	4982 (44.52)	2535 (50.72)	7517 (46.44)
Women	6208 (55.48)	2463 (49.28)	8671 (56.78)
Nationality^a^			
Han	10,438 (93.28)	4741 (94.86)	15,179 (93.77)
Other	752 (6.72)	257 (5.14)	1009 (6.23)
Education			
Junior college or higher	2809 (25.10)	1213 (24.27)	4022 (24.85)
Middle school	6666 (59.57)	3004 (60.10)	9670 (59.74)
Primary school	1343 (12.00)	614 (12.28)	1957 (12.09)
No school	372 (3.32)	167 (3.34)	539 (3.33)
Family income/year (Yuan)^a^			
≤ 5000	988 (8.83)	364 (7.28)	1352 (8.35)
5001–10,000	1505 (13.45)	748 (14.97)	2253 (13.92)
10,001–30,000	5557 (49.66)	2561 (51.24)	8118 (50.15)
≥ 30,000	3140 (28.06)	1325 (26.51)	4465 (27.58)
Smoking status			
Non-smoker	8001 (71.50)	3525 (70.53)	11,526 (71.20)
Smoker	3189 (28.50)	1473 (29.47)	4662 (28.80)
Alcohol consumption			
Non-consumer	8899 (79.53)	3909 (78.21)	12,808 (79.12)
Consumer	2291 (20.47)	1089 (21.79)	3380 (20.88)
Regular exercise^a^			
No	8131 (72.66)	3581 (71.65)	11,712 (72.35)
Yes	3059 (27.34)	1417 (28.35)	4476 (27.65)
Low calorie and low fat			
controlled diet			
No	8418 (75.23)	3766 (75.35)	12,184 (75.27)
Yes	2772 (24.77)	1232 (24.65)	4004 (24.73)
Sugar-sweetened soft drink^a^			
consumption (day per week)			
≤ 1	9466 (84.59)	4505 (90.14)	13,971 (86.30)
2–4	1225 (10.95)	349 (6.98)	1574 (9.72)
≥ 5	499 (4.46)	144 (2.88)	643 (3.97)
BMI (kg/m^2^, mean ± SD)	21.76 ± 2.01	27.43 ± 2.46	23.51 ± 3.39
Family history of hypertension			
No	7645 (68.32)	3405 (68.13)	11,050 (68.26)
Yes	3545 (31.68)	1593 (31.87)	5138 (31.74)
Prehypertension^a^			
No	5462 (48.81)	1339 (26.79)	6801 (42.01)
Yes	5728 (51.19)	3659 (73.21)	9387 (57.99)
Per capita GDP (Yuan)^d^			70,352 (47,639, 100,423)
PD (person/km^2^)^d^			8475 (3824, 12,667)
Air pollutants (μg/m^3^)^d^			
PM_10_			123 (116, 135)
SO_2_			48 (44, 64)
NO_2_			33 (31, 40)
O_3_			50 (41, 63)

^a^Significant difference exists between normal weight and overweight/obese participants by chi-square test or Student’s t-test (age), *p* < 0.05

^b^748 participants were underweight (BMI < 18.5 kg/m^2^)

^c^540 participants were obesity (BMI ≥ 30 kg/m^2^)

^d^Based on values from 11 districts

Table 1 and Additional file 1: Table S2 present the statistics of PM_10_, SO_2_, NO_2_, and O_3_ measured in the 11 districts, which were also compared with the WHO guidelines and Chinese National Ambient Air Quality Standards. The interquartile range (IQR) for PM_10_, SO_2_, NO_2_, and O_3_ were 19, 20, 9, and 22 μg/m^3^, respectively. Both PM_10_ and SO_2_ exceeded WHO guidelines in all the 11 districts, whereas 90.9 and 27.3% of the districts exceeded PM_10_ and SO_2_ levels, as dictated by the Chinese National Ambient Air Quality Standards. All the air pollutants correlated highly with each other with the exception of NO_2_ with O_3_ and SO_2_ (Additional file 1: Table S3). The mean values for per-capita GDP and PD were 73,459 Yuan and 8733 persons per km^2^, respectively, with wide variations across the 11 districts (Table 1; Additional file 1: Table S2).

**Fig. 1:**
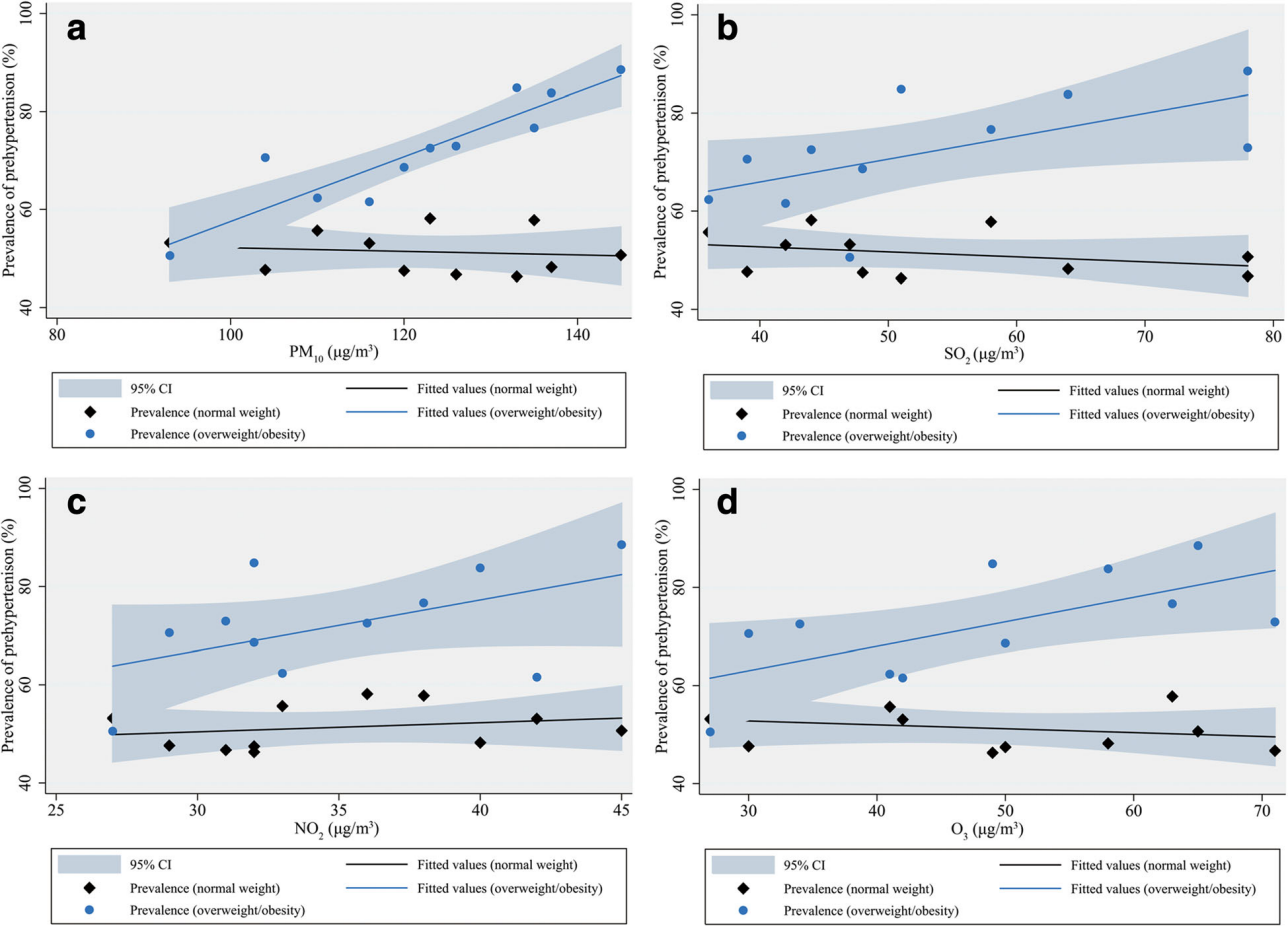
Correlation analysis between air pollution concentrations and prehypertension prevalence in two BMI categories. **a** Scatter plot of prehypertension prevalence versus PM_10_ concentrations (Spearman’s test: normal weight, *r* = − 0.15, *p* = 0.670; overweight/obesity, *r* = 0.909, *p* < 0.001). **b** Scatter plot of prehypertension prevalence versus SO_2_ concentrations (Spearman’s test: normal weight, *r* = − 0.36, *p* = 0.271; overweight/obesity, *r* = 0.729, *p* = 0.011). **c** Scatter plot of prehypertension prevalence versus NO_2_ concentrations (Spearman’s test: normal weight, *r* = 0.325, *p* = 0.328; overweight/obesity, *r* = 0.410, *p* = 0.210). **d** Scatter plot of prehypertension prevalence versus O_3_ concentrations (Spearman’s test: normal weight, *r* = − 0.336, *p* = 0.312; overweight/obesity, *r* = 0.673, *p* = 0.023). Data on air pollution concentrations at the 11 air monitoring stations and prevalence rate of prehypertension among participants living close to each monitoring station were used

Fig. 1 shows the results of Spearman rank correlations between prehypertension rates and the four air pollutants. The prevalence of prehypertension was significantly correlated with PM_10_ (*r* = 0.909, *p* < 0.001), SO_2_ (*r* = 0.729, *p* = 0.011), and O_3_ levels (*r* = 0.673, *p* = 0.023) in overweight participants, but not in participants with normal weight. No significant correlation was found between prehypertension rates and NO_2_ in either the normal weight or overweight participants. We additionally calculated the age- and sex-adjusted prevalence rates of prehypertension according to categories of air pollutants concentrations and BMI, and observed that the prevalence rates of prehypertension were higher in overweight participants than normal weight ones, and the differences were greater among participants exposed at higher air pollutants concentrations (Additional file 1: Table S4).

Table 2 shows the multivariate-adjusted associations of air pollutants with prehypertension from the single-pollutant model. For all participants, the association between each pollutant’s levels and the prevalence of prehypertension was significant among overweight adults (PRRs ranged from 1.14 to 1.20), but not among those with normal weight (PRRs ranged from 0.98 to 1.04). Significant interactions were found for BMI with all four pollutants, with estimated RERIs ranging from 0.41 to 2.17 (All *p*-values ≤0.0041). Further stratifying the analyses by sex, age, GDP, and PD yielded similar results; within each subgroup significant associations between ambient air pollutants and prehypertension were only observed among overweight adults. However, the magnitudes of these significant associations and the estimated RERIs were generally greater in women, those age ≥ 60 years, and participants living in districts with low GDP levels or high PD.

**Table 2:** Associations between prehypertension and air pollutants by BMI categories (single-pollutant model) (*n* = 16,188)

	Normal weight	Overweight	Pollutant*overweight	
Pollutant	PRR (95% CI) ^ab^	PRR (95% CI) ^ab^	RERI (95% CI)	*p*_interaction_ value
Total				
PM_10_	1.01 (0.96, 1.05)	1.20 (1.13, 1.27)	2.17 (1.82, 2.52)	< 0.0001
SO_2_	0.98(0.93, 1.04)	1.14 (1.07, 1.22)	1.98 (1.65, 2.31)	< 0.0001
NO_2_	1.04 (0.98, 1.10)	1.14 (1.06, 1.23)	0.41 (0.13, 0.69)	0.0041
O_3_	1.00 (0.92, 1.06)	1.17 (1.08, 1.27)	1.55 (1.24, 1.86)	< 0.0001
Men				
PM_10_	0.99 (0.93, 1.06)	1.05 (0.96, 1.13)	0.68 (0.13, 1.23)	0.0154
SO_2_	0.97 (0.91, 1.04)	1.03 (0.95, 1.11)	0.56 (0.05, 1.06)	0.0297
NO_2_	1.05 (0.96, 1.13)	1.07 (0.97, 1.18)	0.54 (−0.06, 1.13)	0.0753
O_3_	0.97 (0.89, 1.05)	1.03 (0.93, 1.14)	0.54 (0.01, 1.08)	0.0479
Women				
PM_10_	1.01 (0.94, 1.08)	1.38 (1.26, 1.51)	2.85 (2.35, 3.35)	< 0.0001
SO_2_	0.99 (0.91, 1.07)	1.27 (1.15, 1.41)	2.66 (2.20, 3.13)	< 0.0001
NO_2_	1.02 (0.91, 1.13)	1.21 (1.07, 1.38)	0.33 (−0.03, 0.69)	0.0724
O_3_	1.00 (0.90, 1.12)	1.34 (1.18, 1.52)	1.98 (1.53, 2.42)	< 0.0001
Age < 60 years old				
PM_10_	1.01 (0.96, 1.06)	1.19 (1.12, 1.27)	2.09 (1.72, 2.45)	< 0.0001
SO_2_	0.98 (0.92, 1.04)	1.13 (1.06, 1.21)	1.89 (1.55, 2.22)	< 0.0001
NO_2_	1.04 (0.98, 1.11)	1.13 (1.05, 1.23)	0.42 (0.08, 0.76)	0.0155
O_3_	0.99 (0.92, 1.06)	1.16 (1.06, 1.26)	1.48 (1.16, 1.80)	< 0.0001
Age ≥ 60 years old				
PM_10_	0.99 (0.86, 1.15)	1.24 (1.03, 1.49)	4.23 (2.01, 6.45)	< 0.001
SO_2_	0.98 (0.85, 1.14)	1.17 (0.98, 1.40)	4.68 (2.28, 7.07)	< 0.001
NO_2_	1.00 (0.83, 1.20)	1.15 (0.91, 1.44)	0.36 (−0.78, 1.50)	0.5359
O_3_	0.97 (0.80, 1.17)	1.25 (1.00, 1.57)	3.29 (1.33, 5.24)	< 0.001
GDP < 73,459 Yuan				
PM_10_	1.00 (0.95, 1.04)	1.19 (1.12, 1.27)	2.75 (2.20, 3.30)	< 0.0001
SO_2_	1.00 (0.94, 1.07)	1.21 (1.12, 1.31)	4.06 (3.12, 4.99)	< 0.0001
NO_2_	1.00 (0.94, 1.07)	1.25 (1.15, 1.37)	1.91 (1.47, 2.36)	< 0.0001
O_3_	0.99 (0.92, 1.06)	1.25 (1.14, 1.37)	4.06 (3.12, 4.99)	< 0.0001
GDP ≥ 73,459 Yuan				
PM_10_	1.04 (0.90, 1.21)	1.26 (1.05, 1.52)	1.48 (0.96, 1.99)	< 0.0001
SO_2_	0.93 (0.82, 1.07)	1.00 (0.86, 1.16)	1.25 (0.88, 1.63)	< 0.0001
NO_2_	1.13 (0.99, 1.28)	0.88 (0.76, 1.03)	−1.19 (−1.68, − 0.70)	< 0.0001
O_3_	0.89 (0.67, 1.20)	0.95 (0.70, 1.29)	−0.43 (− 0.90, 0.03)	0.0699
PD < 8733 person/km^2^				
PM_10_	1.00 (0.90, 1.11)	1.22 (1.08, 1.39)	1.24 (0.81, 1.66)	< 0.0001
SO_2_	0.94 (0.86, 1.03)	1.09 (0.98, 1.21)	1.00 (0.70, 1.29)	< 0.0001
NO_2_	1.07 (0.95, 1.20)	1.19 (1.03, 1.39)	0.14 (−0.33, 0.62)	0.5635
O_3_	0.91 (0.80, 1.04)	1.05 (0.91, 1.22)	1.00 (0.70, 1.29)	< 0.0001
PD ≥ 8733 person/km^2^				
PM_10_	1.00 (0.95, 1.06)	1.19 (1.12, 1.28)	4.72 (3.55, 5.88)	< 0.0001
SO_2_	1.02 (0.95, 1.09)	1.18 (1.09, 1.29)	4.72 (3.55, 5.88)	< 0.0001
NO_2_	1.02 (0.93, 1.12)	1.11 (1.00, 1.24)	0.88 (0.35, 1.41)	0.0011
O_3_	1.01 (0.93, 1.09)	1.27 (1.15, 1.41)	5.28 (3.44, 7.12)	< 0.0001

^a^Adjusted for age, sex, race, education, income, smoking, drinking, exercise, diet, sugar intake, family history of hypertension, GDP, and PD; age, sex, GDP, and PD were excluded in stratified analyses by age, sex, GDP, and PD, respectively

^b^PRR was scaled to the interquartile range (IQR) for each pollutant (19 μg/m^3^ for PM_10_, 20 μg/m^3^ for SO_2_, 9 μg/m^3^ for NO_2_, and 22 μg/m^3^ for O_3_)

Pollutant*overweight indicates the interaction of air pollution with overweight

Table 3 shows the associations of air pollutants with SBP and DBP, modified by BMI from the single-pollutant model. For the total sample, all four air pollutants were significantly associated with SBP levels in both overweight and normal weight participants (the exception is NO_2_). However, the increases in SBP levels for every IQR increment of the four pollutants in overweight participants (1.82–4.53 mmHg) were much higher than those in participants with normal weight (0.10–0.61 mmHg). The *p*-values for interactions of all the four pollutants with SBP were < 0.0001. Similar results were observed for DBP. We performed sensitivity analyses by excluding participants who were underweight (Additional file 1: Table S5), hypotensive (Additional file 1: Table S6), or with diabetes mellitus (Additional file 1: Table S7), and the estimates did not substantially change. Stratified analyses showed that the associations between air pollutants and blood pressure levels (mainly in overweight participants) as well as the interactive effects of air pollutants and overweight on BP levels, were generally greater in women, those age ≥ 60 years, and participants living in districts with low GDP levels and/or high PD. For example, the increases in SBP and DBP for every IQR increment of air pollutants were 2.43–5.69 mmHg and 1.20–2.27 mmHg in overweight women, but the corresponding increases in overweight men were 1.17–2.94 mmHg and 0.42–0.97 mmHg, respectively (Table 3).

**Table 3:** Associations between blood pressures and air pollutants by BMI categories (single-pollutant model) (*n* = 16,188)

	Systolic blood pressure				Diastolic blood pressure			
	Normal weight	Overweight	Pollutant*overweight	*p*_interaction_	Normal weight	Overweight	Pollutant*overweight	*p*_interaction_
Pollutant	Estimate (95% CI)^ab^	Estimate (95% CI)^ab^	Estimate (95% CI)^ab^	value	Estimate (95% CI)^ab^	Estimate (95% CI)^ab^	Estimate (95% CI)^ab^	value
Total								
PM_10_	0.49 (0.18, 0.80)	3.93 (3.53, 4.33)	0.73 (0.68, 0.79)	< 0.0001	0.22(0.01, 0.43)	1.37 (1.12, 1.63)	0.50 (0.47, 0.54)	< 0.0001
SO_2_	0.42 (0.12, 0.72)	3.13 (2.74, 3.51)	1.74 (1.61, 1.86)	< 0.0001	0.18 (− 0.02, 0.39)	1.15 (0.91, 1.39)	1.17 (1.08, 1.25)	< 0.0001
NO_2_	0.10 (−0.28, 0.47)	1.82 (1.30, 2.33)	1.19 (1.10, 1.28)	< 0.0001	0.14 (−0.12, 0.39)	0.80 (0.48, 1.12)	0.82 (0.76, 0.88)	< 0.0001
O_3_	0.61 (0.21, 1.01)	4.53 (4.02, 5.05)	2.08 (1.93, 2.23)	< 0.0001	0.36 (0.09, 0.63)	1.63 (1.31, 1.95)	1.39 (1.29, 1.49)	< 0.0001
Men								
PM_10_	0.25 (−0.17, 0.67)	2.21 (1.67, 2.76)	0.65 (0.58, 0.73)	< 0.0001	0.26 (−0.04, 0.56)	0.70 (0.36, 1.03)	0.46 (0.41, 0.51)	< 0.0001
SO_2_	0.27 (−0.14, 0.68)	1.89 (1.37, 2.40)	1.53 (1.36, 1.69)	< 0.0001	0.25 (−0.04, 0.54)	0.65 (0.33, 0.97)	1.03 (0.92, 1.15)	< 0.0001
NO_2_	0.03 (−0.48, 0.55)	1.17 (0.50, 1.84)	1.07 (0.95, 1.19)	< 0.0001	0.11 (−0.25, 0.48)	0.42 (0.01, 0.83)	0.75 (0.67, 0.83)	< 0.0001
O_3_	0.33 (−0.21, 0.88)	2.94 (2.26, 3.63)	1.83 (1.63, 2.03)	< 0.0001	0.52 (0.13, 0.92)	0.97 (0.55, 1.40)	1.23 (1.09, 1.37)	< 0.0001
Women								
PM_10_	0.64 (0.21, 1.08)	5.30 (4.74, 5.87)	0.68 (0.60, 0.76)	< 0.0001	0.19 (−0.10, 0.48)	2.03 (1.65, 2.41)	0.50 (0.45, 0.56)	< 0.0001
SO_2_	0.48 (0.07, 0.90)	4.06 (3.50, 4.62)	1.66 (1.47, 1.84)	< 0.0001	0.14 (− 0.14, 0.41)	1.64 (1.27, 2.01)	1.18 (1.06, 1.30)	< 0.0001
NO_2_	0.12 (− 0.40, 0.64)	2.43 (1.67, 3.19)	1.10 (0.97, 1.23)	< 0.0001	0.10 (− 0.24, 0.45)	1.20 (0.71, 1.68)	0.81 (0.73, 0.90)	< 0.0001
O_3_	0.74 (0.18, 1.31)	5.69 (4.95, 6.43)	1.98 (1.76, 2.21)	< 0.0001	0.23 (−0.15, 0.60)	2.27 (1.78, 2.75)	1.41 (1.26, 1.56)	< 0.0001
Age < 60 years								
PM_10_	0.52 (0.20, 0.84)	3.72 (3.30, 4.14)	0.76 (0.70, 0.82)	< 0.0001	0.28 (0.06, 0.49)	1.38 (1.11, 1.65)	0.50 (0.46, 0.54)	< 0.0001
SO_2_	0.46 (0.15, 0.77)	3.00 (2.59, 3.40)	1.79 (1.66, 1.92)	< 0.0001	0.24 (0.03, 0.45)	1.16 (0.91, 1.42)	1.15 (1.06, 1.24)	< 0.0001
NO_2_	0.08(−0.31, 0.46)	1.69 (1.16, 2.23)	1.24 (1.15, 1.33)	< 0.0001	0.14 (−0.12, 0.41)	0.76 (0.42, 1.09)	0.81 (0.75, 0.88)	< 0.0001
O_3_	0.68 (0.26, 1.09)	4.34 (3.81, 4.88)	2.14 (1.98, 2.30)	< 0.0001	0.42 (0.14, 0.70)	1.64 (1.30, 1.98)	1.37 (1.27, 1.48)	< 0.0001
Age ≥ 60 years								
PM_10_	0.40 (−0.68, 1.49)	5.27 (4.06, 6.48)	0.60 (0.42, 0.79)	< 0.0001	−0.30 (− 0.98, 0.39)	1.35 (0.63, 2.07)	0.47 (0.35, 0.58)	< 0.0001
SO_2_	0.43 (−0.62, 1.48)	3.55 (2.38, 4.72)	1.50 (1.08, 1.93)	< 0.0001	−0.32 (− 0.98, 0.35)	0.89 (0.21, 1.56)	1.11 (0.84, 1.38)	< 0.0001
NO_2_	−0.74 (−2.12, 0.64)	3.04 (1.50, 4.58)	1.01 (0.70, 1.31)	< 0.0001	−0.07 (− 0.94, 0.81)	1.32 (0.45, 2.19)	0.75 (0.56, 0.94)	< 0.0001
O_3_	0.47 (−0.96, 1.89)	5.33 (3.75, 6.91)	1.83 (1.32, 2.34)	< 0.0001	−0.28 (−1.18, 0.62)	1.32 (0.40, 2.25)	1.33 (1.01, 1.65)	< 0.0001
GDP < 73,459 Yuan								
PM_10_	0.40 (0.11, 0.69)	3.48 (3.10, 3.86)	0.75 (0.68, 0.83)	< 0.0001	0.21 (0.02, 0.41)	1.25 (1.01, 1.50)	0.51 (0.46, 0.56)	< 0.0001
SO_2_	0.47 (0.04, 0.90)	4.39 (3.82, 5.00)	1.13 (1.66, 2.00)	< 0.0001	0.24 (−0.06, 0.53)	1.63 (1.26, 1.99)	1.20 (1.08, 1.32)	< 0.0001
NO_2_	0.50 (0.10, 0.90)	4.45 (3.91, 4.99)	1.20 (1.09, 1.32)	< 0.0001	0.30 (0.03, 0.57)	1.61 (1.27, 1.96)	0.82 (0.74, 0.90)	< 0.0001
O_3_	0.54 (0.11, 0.97)	4.78 (4.22, 5.34)	2.40 (2.18, 2.63)	< 0.0001	0.35 (0.05, 0.64)	1.73 (1.37, 2.09)	1.55 (1.40, 1.70)	< 0.0001
GDP ≥ 73,459 Yuan								
PM_10_	0.38 (−0.59, 1.34)	4.07 (2.75, 5.39)	0.71 (0.63, 0.79)	< 0.0001	−0.05 (− 0.70, 0.60)	1.26 (0.43, 2.09)	0.50 (0.44, 0.55)	< 0.0001
SO_2_	0.36 (−0.40, 1.17)	2.71 (1.67, 3.75)	1.62 (1.44, 1.81)	< 0.0001	0.32 (−0.19, 0.83)	0.83 (0.18, 1.48)	1.12 (1.00, 1.24)	< 0.0001
NO_2_	−0.86 (−1.56, − 0.17)	−4.95 (−5.87, −4.03)	1.15 (1.02, 1.28)	< 0.0001	−0.30 (− 0.77, 0.17)	− 1.21 (− 1.80, − 0.63)	0.83 (0.74, 0.92)	< 0.0001
O_3_	0.72 (− 0.99, 2.43)	5.73 (3.37, 8.08)	1.80 (1.60, 2.01)	< 0.0001	0.69 (− 0.47, 1.84)	1.80 (0.33, 3.27)	1.26 (1.13, 1.40)	< 0.0001
PD < 8733 person/km^2^								
PM_10_	0.81 (0.15, 1.47)	5.87 (4.97, 6.76)	0.68 (0.61, 0.75)	< 0.0001	−0.03 (− 0.48, 0.43)	2.21 (1.65, 2.76)	0.50 (0.45, 0.55)	< 0.0001
SO_2_	0.62 (0.14, 1.11)	4.23 (3.58, 4.87)	1.64 (1.48, 1.81)	< 0.0001	0.18 (−0.15, 0.50)	1.48 (1.07, 1.89)	1.16 (1.05, 1.27)	< 0.0001
NO_2_	0.25 (−0.47, 0.97)	2.97 (1.96, 3.98)	1.15 (1.03, 1.27)	< 0.0001	−0.20 (− 0.69, 0.29)	1.30 (0.68, 1.92)	0.85 (0.77, 0.93)	< 0.0001
O_3_	0.82 (0.10, 1.54)	5.81 (4.84, 6.78)	1.84 (1.65, 2.03)	< 0.0001	0.37 (−0.13, 0.86)	2.00 (1.40, 2.61)	1.32 (1.20, 1.45)	< 0.0001
PD ≥ 8733 person/km^2^								
PM_10_	0.44 (0.09, 0.79)	3.62 (3.19, 4.06)	0.81 (0.72, 0.89)	< 0.0001	0.32 (0.08, 0.55)	1.21 (0.93, 1.49)	0.51 (0.45, 0.56)	< 0.0001
SO_2_	0.37 (−0.06, 0.81)	3.53 (2.98, 4.08)	1.89 (1.69, 2.08)	< 0.0001	0.27 (−0.03, 0.56)	1.22 (0.87, 1.57)	1.17 (1.04, 1.30)	< 0.0001
NO_2_	0.14 (−0.26, 0.55)	1.85 (1.31, 2.39)	1.22 (1.09, 1.35)	< 0.0001	0.26 (−0.01, 0.54)	0.76 (0.42, 1.09)	0.78 (0.70, 0.87)	< 0.0001
O_3_	0.55 (0.03, 1.07)	4.78 (4.13, 5.44)	2.53 (2.28, 2.78)	< 0.0001	0.43 (0.08, 0.78)	1.64 (1.23, 2.06)	1.53 (1.36, 1.70)	< 0.0001

^a^Adjusted by age, sex, race, education, income, smoking, drinking, exercise, diet, sugar intake, family history of hypertension, GDP, and PD; age, sex, GDP, and PD were excluded in stratified analyses by age, sex, GDP, and PD, respectively

^b^Estimate was scaled to the interquartile range (IQR) for each pollutant (19 μg/m^3^ for PM_10_, 20 μg/m^3^ for SO_2_, 9 μg/m^3^ for NO_2_, and 22 μg/m^3^ for O_3_)

Pollutant*overweight indicates the interaction of air pollution with overweight

We further estimated associations of air pollutants with blood pressure and prehypertension in two BMI categories using the multi-pollutant model. The results showed that the magnitudes of the associations were only slightly attenuated, and the direction of the associations did not change (Table 4).

**Table 4:** Associations of air pollutants with blood pressures and prehypertension in two BMI categories (multiple-pollutant model)

	Normal weight	Overweight	Pollutant*overweight	*p*_interaction_
Pollutant	Estimate (95% CI)^abc^	Estimate (95% CI)^abc^	Estimate (95% CI)^abd^	value
SBP				
PM_10_	0.21 (− 0.30, 0.73)	2.22 (1.57, 2.88)	2.93 (2.48, 3.39)	< 0.0001
SO_2_	0.16 (− 0.22, 0.55)	1.68 (1.18, 2.17)	2.48 (2.00, 2.97)	< 0.0001
NO_2_	0.20 (−0.27, 0.67)	2.04 (1.44, 2.64)	1.91 (1.34, 2.49)	< 0.0001
O_3_	0.21 (−0.29, 0.71)	2.16 (1.52, 2.80)	2.73 (2.17, 3.28)	< 0.0001
DBP				
PM_10_	0.24 (−0.11, 0.59)	1.07 (0.65, 1.49)	0.99 (0.69, 1.30)	< 0.0001
SO_2_	0.18 (−0.08, 0.44)	0.80 (0.49, 1.12)	0.83 (0.51, 1.16)	< 0.0001
NO_2_	0.22 (−0.10, 0.54)	0.98 (0.59, 1.36)	0.72 (0.34, 1.11)	0.0002
O_3_	0.23 (−0.11, 0.57)	1.04 (0.63, 1.45)	0.91 (0.54, 1.28)	< 0.0001
Prehypertension prevalence				
PM_10_	0.99 (0.92, 1.06)	1.18 (1.09, 1.27)	2.40 (1.85, 2.95)	< 0.0001
SO_2_	0.99 (0.93, 1.04)	1.14 (1.07, 1.22)	2.12 (1.71, 2.53)	< 0.0001
NO_2_	1.02 (0.95, 1.09)	1.12 (1.03, 1.21)	0.51 (0.18, 0.83)	0.0021
O_3_	0.99 (0.92, 1.06)	1.18 (1.08, 1.27)	1.53 (1.21, 1.84)	< 0.0001

^a^Adjusted by age, sex, race, education, income, smoking, drinking, exercise, diet, sugar intake, family history of hypertension, gross domestic product, population density, and residuals generated from regression analyses for highly correlated pollutants (PM_10_ vs. SO_2_, PM_10_ vs. NO_2_, PM_10_ vs. O_3_, and SO_2_ vs. O_3_)

^b^Estimate was scaled to the interquartile range (IQR) for each pollutant (19 μg/m^3^ for PM_10_, 20 μg/m^3^ for SO_2_, 9 μg/m^3^ for NO_2_, and 22 μg/m^3^ for O_3_)

^c^Estimate for SBP and DBP was regression coefficient (β), and for prehypertension prevalence were PRR

^d^Interactive estimate for SBP and DBP with body mass index categories was regression coefficient (β) and its corresponding 95% CI, and for PRR was the relative excessive risk due to interaction (RERI) and its corresponding 95% CI

Pollutant*overweight indicates the interaction of air pollution with overweight

## Discussion

In this large cross-sectional study of 15,477 Chinese adults, we found that being overweight modified the associations of long-term exposure to ambient air pollution (PM_10_, SO_2_, NO_2_, and O_3_) with prehypertension and arterial blood pressure in adults. The observed associations were mainly significant among overweight participants, and the magnitude of these significant associations were generally greater among women, those age ≥ 60 years, or participants living in districts with lower income levels or with higher PD. Overall, these results suggest that overweight may appreciably modify the susceptibility to pro-hypertensive effects of airborne pollutants, particularly for women, the elderly, or those living in areas with lower income levels or higher PD.

To our knowledge, this is the first attempt to explore the interactive effects of ambient air pollutants and BMI on prehypertension. Thus, it is difficult to directly compare our present findings with those from other studies. However, in a systematic Medline search, we found that four epidemiological studies have considered BMI as an effect modifier of the association between ambient air pollution and hypertension and blood pressure. In the Nurses’ Health Study, a population-based prospective cohort in the US, Zhang et al. examined the role of chronic exposures to PM_2.5_, PM_2.5–10_, PM_10_, and proximity to major roadways as risk factors for incident hypertension in 74,880 females aged 30–50 years, and explored whether some lifestyle and exposure related factors (e.g. age, obesity, diabetes, etc.) acted as potential association modifiers. The results indicated that each 10 μg/m^3^ increase in PM_2.5_, PM_2.5–10_ and PM_10_ was associated with an increased risk of incident hypertension, and higher risks were observed for obese women (hazard ratio (HR) 24-month average PM_10_: 1.07, 95% CI: 1.04–1.12; HR PM_2.5_: 1.15, 95% CI: 1.07–1.23; HR PM_2.5–10_: 1.13; 95% CI: 1.07–1.19) [27]. Using part of the Healthy Environments Partnership Study data, Kannan et al. evaluated the acute effect of exposure to PM_2.5_ on blood pressure among 348 American adults aged ≥25 years old. This study reported associations between PM_2.5_ exposure and elevated pulse pressure among obese individuals. For example, an increment of 10 μg/m^3^ in daily PM_2.5_ was associated with a 2.55 mmHg increase in pulse pressure at lag 3 in obese individuals, but a 0.09 mmHg decrease in pulse pressure was observed in the non-obese individuals [14].

Two other relevant studies were previously conducted by our own research group. One was the Seven Northeastern Cities (SNEC) study [16]. In that study, we examined the synergistic effects of ambient air pollution exposure (PM_10_, SO_2_, NO_2_, and O_3_) and obesity on hypertension and blood pressure in 9354 children, and observed that the association between exposure to each pollutant and prevalent hypertension was strongest in obese children (OR ranged from 1.16 to 2.91), less strong in overweight children (OR ranged from 1.12 to 2.05), and weakest in normal weight children (OR ranged from 0.82 to 1.21). Additionally, exposure to all ambient air pollutants except NO_2_ was associated with higher arterial blood pressures and the magnitude of the association increased with BMI. The other was the 33CCHS, a larger population-based cross-sectional study of ambient air pollution and adult health in the same province. In that study, we tested the same hypothesis in 24,845 adults aged ≥18 years old and obtained similar results [15].

Although the four studies mentioned above [14–16, 27] were conducted in different geographical areas with relevant variations in population characteristics, pollutant sources and components, timing of exposure, pollutant concentrations, and exposure assessment, these studies concordantly demonstrated the interaction of overweight and obesity on air pollution effects on hypertension and/or blood pressure. These prior findings were roughly comparable to our present results and provide important support for our hypothesis that overweight modifies the hazardous effects of air-borne pollutants on prehypertension and arterial blood pressure. Since both overweight/obesity and prehypertension are major risk factors for cardiovascular diseases, our present findings combined with those prior studies [14–16, 27], thus have significant public health implications in the form of additional evidence that government and individuals should take urgent strategies to reduce exposure to air pollutants, especially for people who have higher BMI.

Although the mechanism underlying the synergistic effects of overweight/obesity and ambient airborne pollutants on BP and prehypertension is not well understood, there are several candidates. Foremost among these is that overweight/obesity is associated with dysfunction of the adipose tissue, which would lead to activation of the renin-angiotensin-aldosterone system, oxidative stress, and chronic vascular inflammation. These processes may ultimately cause prehypertension [12]. Ambient air pollutants could contribute to the development of prehypertension through promoting systemic inflammation and oxidative stress, instigating autonomic dysfunction, and triggering vascular endothelial dysfunction [7]. Inflammation, for example, is a shared risk factor of air pollution and overweight/obesity in increasing the probability of prehypertension [7, 12]. Additionally, laboratory evidence has demonstrated that exposure to PM_2.5_ can amplify adipose inflammation in obese mice [13]. Prehypertension is a chronic inflammatory state aggravated by factors promoting inflammation at the level of vasculature and adipose tissue [28, 29]. Therefore, it seems reasonable to suggest that overweight participants might be more susceptible to the inflammatory effects of ambient air pollutants, leading to a higher prevalence of elevated blood pressure, including prehypertension. This would partially explain the interaction observed in our present study. Furthermore, in our analysis the participants in the normal weight group are normotensive and non-medicated individuals, thus the healthy survivor bias is possible in these participants and it might be a possible reason for lower susceptibility of the normal weight group to air pollution.

In stratified analyses by sex, we found stronger interactions between overweight/obesity and air pollution on prehypertension and blood pressure in women than in men. Our results were in agreement with previous studies by Dong et al. [16] and Zhao et al. [15] that detected stronger synergistic effects of obesity and air pollution on hypertension and arterial blood pressures in girls and adult women. Our results were also parallel to studies on gender-specific associations between air pollution and other health outcomes [20, 30, 31]. Qin et al. [20] reported that the interactions of PM_10_, SO_2_, NO_2_, and O_3_ with obesity on cardiovascular diseases and stroke were only obtained in women. Franklin et al. [30] found that air pollution was a stronger predictor of death among females than among males. Moreover, Kan et al. [31] observed that women were more susceptible to air pollution exposure in a time-series analysis study in Shanghai, China. Sex-specific lifestyles (e.g. more male smokers than female smokers in China) [32], biological explanations (e.g. smaller airways for females) [33], and greater deposition of particles in the lung [34, 35] might partially explain the gender-specific effects.

When stratified by age, stronger synergistic effects of overweight/obesity and air pollution were also observed among older individuals, especially for the prevalence of prehypertension and SBP levels. There has been accumulating epidemiological evidence of the modification of age on exposure to air pollution and health. In accord with our findings, Baumgartner et al. [36] reported that among women > 50 years of age the increases in SBP and DBP per 1-log-μg/m^3^ increase in PM_2.5_ were 4.1 mmHg (95% CI: 1.5–6.6) and 1.8 mmHg (95% CI: 0.4–3.2) mm Hg. However, in women aged 20–50 years old the associations were not statistically significant. Our previous work also found that the associations of PM_10_, SO_2_, and O_3_ with hypertension were stronger among participants ≥65 years of age than in participants ≤55 or 55–65 years of age [22]. The higher estimated effects among older individuals in these prior studies may be attributable to long-term oxidative stress and accumulated systemic inflammation resulting from lifetime PM exposure [36]. On the contrary, Zhang et al. [27] observed that the effects of PM_2.5_, PM_2.5–10_, and PM_10_ on incident hypertension were stronger among women under 65 years of age, and Dvonch et al. [37] reported stronger estimated effects of PM on blood pressure in women < 50 years of age. The authors speculated that older participants were more likely to take blood pressure medication, which might result in a dampening of the effect of PM on blood pressure in this age group. The other explanation is the differences in time-activity patterns between older and younger participants [27, 37].

In addition, the results suggested that the interactive effects were stronger among people living in areas with lower income levels and/or with higher PD. To the best of our knowledge, no prior study explored whether area-level SES modified synergistic effects of air pollution and overweight/obesity on blood pressures. However, several epidemiological studies have reported that people with lower SES showed stronger associations between air pollutants and other health outcomes [38, 39]. Nevertheless, our findings are not unexpected as people living in areas with lower SES and/or higher PD are more likely to be exposed to higher levels of air pollutants [40]. In addition, people with lower SES usually have poorer health status or poor access to health care services [41].

Despite its assets, this study has several limitations. First, owing to the cross-sectional study design, the findings cannot be used to infer any cause-and-effect relationship, but can help generate a hypothesis. Second, since hypertensive individuals (who are usually older adults) were excluded from the study sample, the healthy survivor bias was possible and the effects of air pollutants on blood pressure might have been underestimated. Therefore, any extrapolations to the general population should be made with caution. Third, there may be recall bias since we relied on a questionnaire to collect exposure data (e.g. smoking and drinking). Fourth, though we attempted to control for a wide range of important variables such as physical activity, individual-level SES, and dietary habits, other relevant potential confounders like food environment, walkability, occupational data, and household air pollution were not collected in this study. In addition, we did not collect detailed data on diet and smoking. High sodium intakes, for example, a main risk factor for high BP, were not included and controlled. Smoking status was only grouped into current smokers and non-smokers, whereas smoking intensity and duration were not considered, thus a covariate misclassification was possible. The residual confounding caused by unmeasured covariate (dietary sodium) and covariate misclassification (smoking status) might have caused an overestimation of the effects of air pollutants (data not shown). Moreover, data on secondary hypertension or medications that have bearing on BP or adiposity were also not available. Fifth, potential misclassification of smoking, drinking, and exercising could exist due to the dichotomous responses (simply yes or no, rather than continuous). In addition, exercise might be a mediator, as more polluted areas could make the environment less appealing setting for outdoor physical activity. Alternatively, people with pre-hypertension might have modified their lifestyle as part of the treatment plan. Sixth, we only assessed blood pressure levels with standard protocols of repeated measurements at one point in time, which is limited in representing long-term patterns of blood pressures. Seventh, the 3-year average concentrations of PM_10_, SO_2_, NO_2_, and O_3_ were calculated from daily measurements of the existing monitors, which only reflected the background air pollution levels, thus likely underestimating the air pollutant exposure levels and overestimating its pro-hypertensive effects. Eighth, in addition to environmental factors, the development of prehypertension is also influenced by genetic factors. The design of our study, however, could not separate genetic and environmental influences. Finally, in urban settings, air pollutant exposures often go together with noise (especially traffic noise), which potentially confounds the effects of air pollutions on BP [42]. While it would be better to adjust for the effects of noise in our analysis, unfortunately, no noise data was available to this study.

## Conclusion

In conclusion, our results found that overweight may enhance the effects of ambient air pollutants on prehypertension, and the modifying effects were more apparent among women, those age ≥ 60 years, or participants who living in areas with lower GDP levels or higher PD. Considering the existence of both the current overweight/obesity epidemic and the high ambient air pollution levels in China, there is an urgent need for government to develop effective prevention and intervention policies to protect people from suffering adverse health effects of ambient air pollution, especially among those having higher BMI.

## Additional file

Additional file 1: Outlining baseline characteristics of the study population, air pollutants exposures, and additional modeling details. Supplementary methods. explanation of the air pollution data. **Table S1.** Characteristics of the study participants and non-participants. **Table S2.** Three-year average concentrations of air pollutants and area-level GDP and PD in 11 districts. **Table S3.** Pair-wise correlations of air pollutants. **Table S4.** Age- and sex-adjusted prevalence rate of prehypertension in relation to categories of air pollutants concentrations and BMI. **Table S5.** Associations between air pollutants and blood pressures in two BMI categories after excluding participants who were underweight. **Table S6.** Associations between air pollutants and blood pressures in two BMI categories after excluding hypotensive participants. **Table S7.** Associations between air pollutants and blood pressures in two BMI categories after excluding participants with diabetes mellitus. (DOCX 61 kb)

## Abbreviations

33CCHS: The 33 Communities Chinese Health Study
BMI: Body mass index
CI: Confidence interval
DBP: Diastolic blood pressure
GDP: Gross domestic product
HR: Hazard ratio
IQR: Interquartile range
JNC-7: The Seventh Report of the Joint National Committee on Prevention, Detection, Evaluation, and Treatment of High Blood Pressure
NAAQS: National Ambient Air Quality Standards of China
NO_2_: Nitrogen dioxide
O_3_: Ozone
PD: Population density
PM_10_: Particulate matter with an aerodynamic diameter ≤ 10 μm
PRR: Prevalence rate ratio
QT interval: The time between the start of the Q wave and the end of the T wave in the heart’s electrical cycle
SBP: Systolic blood pressure
SD: Standard deviation
SES: Socioeconomic status
SO_2_: Sulfur dioxide
US: United States
WHO: World Health Organization
