# Supplementary material for: Overweight modifies the association between long-term ambient air pollution and prehypertension in Chinese adults: the 33 Communities Chinese Health Study
Source: Environ Health. 2018 Jun 28;17:57. doi: 10.1186/s12940-018-0401-2 (PMC6022431; doi:10.1186/s12940-018-0401-2)
Supplement: Supplementary file 1 — Outlining baseline characteristics of the study population, air pollutants exposures, and additional modeling details. Supplementary methods. explanation of the air pollution data. Table S1. Characteristics of the study participants and non-participants. Table S2. Three-year average concentrations of air pollutants and area-level GDP and PD in 11 districts. Table S3. Pair-wise correlations of air pollutants. Table S4. Age- and sex-adjusted prevalence rate of prehypertension in relation to categories of air pollutants concentrations and BMI. Table S5. Associations between air pollutants and blood pressures in two BMI categories after excluding participants who were underweight. Table S6. Associations between air pollutants and blood pressures in two BMI categories after excluding hypotensive participants. Table S7. Associations between air pollutants and blood pressures in two BMI categories after excluding participants with diabetes mellitus. (DOCX 61 kb) [file 12940_2018_401_MOESM1_ESM.docx]

**Supplemental Material**

**Overweight modifies the association between long-term ambient air pollution and prehypertension in Chinese adults: the 33 Communities Chinese Health Study**

**Authors**

Bo-Yi Yang^1^, Zhengmin (Min) Qian^2^, Michael G. Vaughn^3^, Steven W. Howard^4^, John Phillip Pemberton^2^, Huimin Ma^5^, Duo-Hong Chen^6^, Li-Wen Hu^1^, Xiao-Wen Zeng^1^, Chuan Zhang^1^, Yan-Peng Tian^1^, Min Nian^1^, Xiang Xiao^1^, and Guang-Hui Dong^1,*^

**Affiliations**

1. Guangzhou Key Laboratory of Environmental Pollution and Health Risk Assessment; Guangdong Provincial Engineering Technology Research Center of Environmental and Health risk Assessment); Department of Preventive Medicine, School of Public Health, Sun Yat-sen University, Guangzhou 510080, China. Email: yangby23@mail.sysu.edu.cn (Bo-Yi Yang); foxhlw@126.com (Li-Wen Hu); zxw63@mail.sysu.edu.cn (Xiao-Wen Zeng); zhang3tiao@126.com (Chuan Zhang); tianyanp@mail2.sysu.edu.cn (Yan-Peng Tian); tingyuyan@126.com (Min Nian); xiaox37@mail2.sysu.edu.cn (Xiang Xiao).
2. Department of Epidemiology, College for Public Health and Social Justice, Saint Louis University, Saint Louis 63104, USA. Email: zqian2@slu.edu (Zhengmin (Min) Qian); [pembertonjr@slu.edu](mailto:pembertonjr@slu.edu) (John Phillip Pemberton).
3. School of Social Work, College for Public Health and Social Justice, Saint Louis University, Saint Louis 63104, USA. Email: [mvaughn9@slu.edu](mailto:mvaughn9@slu.edu) (Michael G. Vaughn).
4. Department of Health Management & Policy, College for Public Health & Social Justice, Saint Louis University. 3545 Lafayette Avenue, Saint Louis, MO 63104. Email: [Steven.Howard@slu.edu](mailto:Steven.Howard@slu.edu) (Steven W. Howard).
5. State Key Laboratory of Organic Geochemistry and Guangdong Key Laboratory of Environmental Protection and Resources Utilization, Guangzhou Institute of Geochemistry, Chinese Academy of Sciences, Guangzhou 510640, China. Email: [mahuimin@gig.ac.cn](mailto:mahuimin@gig.ac.cn) (Huimin Ma).
6. Guangdong Environmental Monitoring Center, State Environmental Protection Key Laboratory of Regional Air Quality Monitoring, Guangdong Environmental Protection Key Laboratory of Atmospheric Secondary Pollution, Guangzhou 510308, China. Email: [13710967699@139.com](mailto:13710967699@139.com) (Duo-Hong Chen).

***Correspondence**

Guang-Hui Dong, MD, PhD, Professor, Department of Preventive Medicine, School of Public Health, Sun Yat-sen University, 74 Zhongshan 2^nd^ Road, Yuexiu District, Guangzhou 510080, PR China. Phone: +86-20-87333409; Fax: +86-20-87330446. Email: [donggh5@mail.sysu.edu.cn](mailto:donggh5@mail.sysu.edu.cn)

**Summary**

Being overweight may enhance the effects of ambient air pollution on prehypertension and blood pressure in Chinese adults.

**Table of Contents**

**Supplementary methods:** explanation of the air pollution data

**Table S1** Characteristics of the study participants and non-participants

**Table S2** Three-year average concentrations of air pollutants and area-level GDP and PD in 11 districts

**Table S3** Pair-wise correlations of air pollutants

**Table S4** Age- and sex-adjusted prevalence rate of prehypertension in relation to categories of air pollutants concentrations and BMI

**Table S5** Associations between air pollutants and blood pressures in two BMI categories after excluding participants who were underweight (n = 15,440)

**Table S6** Associations between air pollutants and blood pressures in two BMI categories after excluding hypotensive participants (n = 15,804)

**Table S7** Associations between air pollutants and blood pressures in two BMI categories after excluding participants with diabetes mellitus (n = 15,829)

**Supplementary methods: explanation of the air pollution data**

The operation of the monitoring stations has strictly followed the quality assurance/quality control (QA/QC) procedure set by the State Environmental Protection Administration of China (SEPAC, 1992). The environmental monitoring centers in each of the three cities conducted regular performance audits and precision checks on the air-monitoring equipment. Quarterly performance audits are conducted to assess data accuracy on PM_10_, SO_2_, NO_2_, and O_3_ monitoring systems.

**1)** **The calculation method**

The calculation method is performed according to Chinese National standards (GB8170-87). The unit of monitored pollutants is mg/m^3^, accurate to the third decimal (0·000). The units can also be expressed as μg/m^3^, depending on the pollutant's concentration. For concentrations that were too low to be measured, half of the lowest checking limit of the equipment will be used as the measured value.

**2)** **Outliers**

When the measured concentration is too low (e.g. background value), a negative value can be obtained because of the zero drift of the monitor. There is no physical meaning to this value. This negative value can be regarded as a value of "unable to measure."

For the monitoring station with an automatic calibration system, if equipment zero drift/span drift exceeds the control range during the period of zero/span calibration, the data from the time it becomes out of control until the equipment is recovered should be regarded as invalid data. The data cannot be used statistically.

The data during the period of zero calibration/span calibration should be regarded as invalid data. It cannot be used statistically, but a flag should be made on these data and the records stored as evidence.

When values are missing because of a loss of power, any data received by the central control station during the period of the loss of power should be regarded as invalid data. The period of loss of power should be counted at the start of power outage until complete warm-up of equipment. The data cannot be used statistically.

Because pollutant concentrations change over time and change slowly, there should be no swift change in pollutant concentration in the results of normal monitoring. Either a swift change or no change indicates that there is an equipment problem. The problem should be identified, and the data between the start of the problem to recovery should be regarded as outliers. These data cannot be used statistically.

**3) Statistics of monitoring data**

One-time value

The central control station uses an average of 15 minutes of pollutant concentrations measured at the branch station as a one-time value. The central control modifies this value and judges whether this value is an outlier using the report software.

One-hour mean value

At least 75% of the one-time values should be used to calculate the one-hour average mean value. One-hour average mean value is calculated by averaging all the valid one-time values within one hour.

Daily average mean

For PM_10_ at least 12 valid hourly mean values are needed to calculate the daily mean value (using the calendar as the valid time frame), using all available valid hourly mean values. For SO_2_ and NO_2_ at least 18 hourly mean values everyday are needed to calculate valid daily mean value (using the calendar as the valid time frame). For O_3_, at least six hourly concentrations of O_3_ per day are needed for calculating the 8-hour average concentration of O_3_. All of the valid hourly mean values are used to calculate the daily mean. (National Environmental Air Quality Standard GB3095-2012)

Monthly mean values are the arithmetic means of all valid daily mean values within the month. Seasonal mean values are the arithmetic means of all valid daily mean values within the season. Yearly mean values are the arithmetic means of all valid daily mean values within the year. District daily mean values are calculated using the monthly mean value, the seasonal mean value, and the yearly mean value from the available stations in the district.

**Table S1** Characteristics of the study participants and non-participants

| Characteristics | Study participants | Non-participants | Total |
| --- | --- | --- | --- |
|  | (n =16,188) | (n =8657) | ( n = 24,845) |
| Age (years, mean ± SD)^a^ | 42.31 ± 12.75 | 51.74 ± 12.10 | 45.59 ± 13.31 |
| Sex^a^ |  |  |  |
| Men | 7517 (46.4) | 5144 (59.4) | 12,661 (51.0) |
| Women | 8671 (56.8) | 3513 (40.6) | 12,184 (49.0) |
| Nationality^a^ |  |  |  |
| Han | 15,179 (93.8) | 8291 (95.8) | 23,470 (94.5) |
| Others | 1009 (6.2) | 366 (4.2) | 1375 (5.5) |
| Education^a^ |  |  |  |
| Junior college or higher | 4022 (24.9) | 1453 (16.8) | 5475 (22.0) |
| Middle school | 9670 (59.7) | 5263 (60.8) | 14,933 (60.1) |
| Primary school | 1957 (12.1) | 1489 (17.2) | 3446 (13.8) |
| No school | 539 (3.3) | 452 (5.2) | 991 (4.0) |
| Family income per year^a^ |  |  |  |
| ≤5000 Yuan | 1352 (8.4) | 872 (10.1) | 2224 (9.0) |
| 5000-10000 Yuan | 2253 (13.9) | 1284 (14.8) | 3537 (14.2) |
| 10000-30000 Yuan | 8118 (50.2) | 4230 (48.9) | 12,348 (49.7) |
| ≥30000 Yuan | 4465 (27.6) | 2271 (26.2) | 6736 (27.1) |
| Smoking status^a^ |  |  |  |
| Nonsmoker | 11,526 (71.2) | 6017 (69.5) | 17,543 (70.6) |
| Smoker | 4662 (28.8) | 2640 (30.5) | 7302 (29.4) |
| Alcohol consumption^a^ |  |  |  |
| Nondrinker | 12,808 (79.1) | 6274 (72.5) | 19,082 (76.8) |
| Drinking | 3380 (20.9) | 2383 (27.5) | 5763 (23.2) |
| Exercise^a^ |  |  |  |
| No | 11,712 (72.4) | 5486 (63.4) | 17,198 (69.2) |
| Yes | 4476 (27.6) | 3171 (36.6) | 7647 (30.8) |
| Control diet with low calories and low fat |  |  |  |
| No | 12,184 (75.3) | 6473 (74.8) | 18,657 (75.1) |
| Yes | 4004 (24.7) | 2184 (25.2) | 6188 (24.9) |
| Sugar-sweetened soft drink^a^ |  |  |  |
| ≤1 day per week | 13,971 (86.3) | 7968 (92.0) | 21,939 (88.3) |
| 2-4 days per week | 1574 (9.7) | 400 (4.6) | 1974 (8.0) |
| ≥5 days per week | 643 (4.0) | 289 (3.3) | 932 (3.8) |
| Body mass index^a^ |  |  |  |
| <18.5 kg/m^2^ | 748 (4.6) | 64 (0.7) | 812 (3.3) |
| 18.5-24.9 kg/m^2^ | 10,300 (63.6) | 3250 (37.6) | 13,550 (54.5) |
| 25-30 kg/m^2^ | 4600 (28.4) | 4448 (51.4) | 9048 (36.4) |
| ≥30 kg/m^2^ | 540 (3.3) | 895 (10.3) | 1435 (5.8) |
| Family history of hypertension^a^ |  |  |  |
| No | 11,050 (68.3) | 4562 (52.7) | 15,612 (62.8) |
| Yes | 5138 (31.7) | 4095 (47.3) | 9233 (37.2) |

SD indicates standard deviation.

^a^Significant difference exists between study participants and non-participants by chi-square test or Student’s t-test, *p* < 0.05.

**Table S2** Three-year average concentrations of air pollutants and area-level GDP and PD in 11 districts

| City | PM_10_, µg/m^3^ | SO_2_, µg/m^3^ | NO_2_, µg/m^3^ | O_3_, µg/m^3^ | GDP (Yuan) | PD (person/km^2^) |
| --- | --- | --- | --- | --- | --- | --- |
| Shenyang |  |  |  |  |  |  |
| District 1 | 133 | 51 | 32 | 49 | 107,564 | 11,424 |
| District 2 | 123 | 44 | 36 | 34 | 68,178 | 7842 |
| District 3 | 116 | 42 | 42 | 42 | 99,452 | 14,517 |
| District 4 | 145 | 78 | 45 | 65 | 47,639 | 14,050 |
| District 5 | 135 | 58 | 38 | 63 | 126,772 | 450 |
| Anshan |  |  |  |  |  |  |
| District 1 | 126 | 78 | 31 | 71 | 100,423 | 2014 |
| District 2 | 137 | 64 | 40 | 58 | 70,352 | 8462 |
| District 3 | 120 | 48 | 32 | 50 | 74,266 | 8475 |
| Jinzhou |  |  |  |  |  |  |
| District 1 | 104 | 39 | 29 | 30 | 25,500 | 12,667 |
| District 2 | 110 | 36 | 33 | 41 | 62,342 | 3824 |
| District 3 | 93 | 47 | 27 | 27 | 25,561 | 12,333 |
| IQR^a^ | 19 | 20 | 9 | 22 | - | - |
| NAAQS^b^ | 100 | 60 | 40 | 160 | - | - |
| % of >NAAQS | 90.9 | 27.3 | 18.2 | 0.0 | - | - |
| WHO guideline^c^ | 20 | 20 | 40 | 100 | - | - |
| % of >WHO guideline | 100.0 | 100.0 | 18.2 | 0.0 | - | - |

GDP indicates gross domestic product; IQR, interquartile range; NAAQS, National Ambient Air Quality Standards of China; NO_2_, nitrogen dioxides; NS, national standard; O_3_, ozone; PD, population density; PM_10_, particle with aerodynamic diameter ≤10 µm; SD, standard deviation; SO_2_, sulfur dioxide; and WHO, World Health Organization.

^a^range from 25th to 75th percentile of district-specific concentrations.

^b^National ambient air quality standard of China.

^c^WHO 2005 air quality guidelines

**Table S3** Pair-wise correlations of air pollutants

| Air pollutants | Spearman correlation coefficients (*p* value) | | | |
| --- | --- | --- | --- | --- |
|  | PM_10_ | SO_2_ | NO_2_ | O_3_ |
| PM_10_ (µg/m^3^) | 1.00 | 0.81 (0.003) | 0.65 (0.030) | 0.81 (0.002) |
| SO_2_ (µg/m^3^) |  | 1.00 | 0.25 (0.456) | 0.84 (0.001) |
| NO_2_ (µg/m^3^) |  |  | 1.00 | 0.45 (0.164) |
| O_3_ (µg/m^3^) |  |  |  | 1.00 |

Abbreviations: NO_2_, nitrogen dioxides; O_3_, ozone; PM_1.0_, particle with aerodynamic diameter ≤1.0 µm; PM_2.5_, particle with aerodynamic diameter ≤2.5 µm; PM_10_, particle with aerodynamic diameter ≤10 µm; and SO_2_, sulfur dioxide.

**Table S4** Age- and sex-adjusted prevalence rate of prehypertension in relation to categories of air pollutants categories and BMI

| Air pollutants concentrations | BMI categories | Prevalence rate of prehypertension |
| --- | --- | --- |
| High PM_10_ (≥123 µg/m^3^) |  |  |
|  | Normal weight | 56.97% |
|  | Overweight | 60.17% |
| Low PM_10_ (<123 µg/m^3^) |  |  |
|  | Normal weight | 57.44% |
|  | Overweight | 59.23% |
| High SO_2_ (≥48 µg/m^3^) |  |  |
|  | Normal weight | 56.62% |
|  | Overweight | 60.09% |
| Low SO_2_ (<48 µg/m^3^) |  |  |
|  | Normal weight | 57.97% |
|  | Overweight | 59.34% |
| High NO_2_ (≥33 µg/m^3^) |  |  |
|  | Normal weight | 57.57% |
|  | Overweight | 60.04% |
| Low NO_2_ (<33 µg/m^3^) |  |  |
|  | Normal weight | 56.73% |
|  | Overweight | 59.43% |
| High O_3_ (≥50 µg/m^3^) |  |  |
|  | Normal weight | 56.56% |
|  | Overweight | 60.01% |
| Low O_3_ (<50 µg/m^3^) |  |  |
|  | Normal weight | 57.78% |
|  | Overweight | 59.55% |

BMI, indicates body mass index; NO_2_, nitrogen dioxide; O_3_, ozone; PM_10_, particle with aerodynamic diameter ≤10 µm; and SO_2_, sulfur dioxide.

**Table S5** Associations between air pollutants and blood pressures in two BMI categories after excluding participants who were underweight (n = 15,440)

|  | Systolic blood pressure | | |  |  | | Diastolic blood pressure | |  |
| --- | --- | --- | --- | --- | --- | --- | --- | --- | --- |
|  | Normal weight | Overweight | Pollutant*overweight |  |  | Normal weight | Overweight | Pollutant*overweight |  |
| Pollutant | Estimate (95% CI)^ab^ | Estimate (95% CI)^ab^ | Estimate (95% CI)^ab^ | *P*-value |  | Estimate (95% CI)^ab^ | Estimate (95% CI)^ab^ | Estimate (95% CI)^ab^ | *P*-value |
| Total |  |  |  |  |  |  |  |  |  |
| PM_10_ | 0.46 (0.14-0.77) | 3.93 (3.53-4.33) | 0.70 (0.64-0.75) | <0.0001 |  | 0.24 (0.03-0.46) | 1.37 (1.12-1.63) | 0.48 (0.45-0.52) | <0.0001 |
| SO_2_ | 0.38 (0.07-0.68) | 3.13 (2.74-3.51) | 1.66 (1.54-1.79) | <0.0001 |  | 0.18 (-0.02-0.39) | 1.15 (0.91-1.39) | 1.12 (1.03-1.20) | <0.0001 |
| NO_2_ | 0.10 (-0.28-0.48) | 1.82 (1.30-2.33) | 1.14 (1.05-1.23) | <0.0001 |  | 0.17 (-0.09-0.42) | 0.80 (0.48-1.12) | 0.79 (0.73-0.85) | <0.0001 |
| O_3_ | 0.54 (0.13-0.94) | 4.54 (4.02-5.05) | 1.99 (1.84-2.14) | <0.0001 |  | 0.36 (0.08-0.64) | 1.63 (1.31-1.95) | 1.34 (1.24-1.44) | <0.0001 |

CI, indicates confidence interval; NO_2_, nitrogen dioxide; O_3_, ozone; OR, odds ratio; PM_10_, particle with aerodynamic diameter ≤10 µm; and SO_2_, sulfur dioxide.

^a^Adjusted by age, sex, race, education, income, smoking, drinking, exercise, diet, sugar intake, family history of hypertension, GDP, and PD.

^b^Estimate was scaled to the interquartile range (IQR) for each pollutant (19 µg/m^3^ for PM_10_, 20 µg/m^3^ for SO_2_, 9 µg/m^3^ for NO_2_, and 22 µg/m^3^ for O_3_).

**Table S6** Associations between air pollutants and blood pressures in two BMI categories after excluding hypotensive participants (n = 15,804)

|  | Systolic blood pressure | | |  |  | | Diastolic blood pressure | |  |
| --- | --- | --- | --- | --- | --- | --- | --- | --- | --- |
|  | Normal weight | Overweight | Pollutant*overweight |  |  | Normal weight | Overweight | Pollutant*overweight |  |
| Pollutant | Estimate (95% CI)^ab^ | Estimate (95% CI)^ab^ | Estimate (95% CI)^ab^ | *P*-value |  | Estimate (95% CI)^ab^ | Estimate (95% CI)^ab^ | Estimate (95% CI)^ab^ | *P*-value |
| Total |  |  |  |  |  |  |  |  |  |
| PM_10_ | 0.50 (0.18-0.78) | 3.81 (3.42-4.20) | 0.69 (0.64-0.74) | <0.0001 |  | 0.20 (0.00-0.40) | 1.29 (1.05-1.54) | 0.46 (0.43-0.50) | <0.0001 |
| SO_2_ | 0.41 (0.12-0.70) | 3.05 (2.67-3.43) | 1.65 (1.52-1.77) | <0.0001 |  | 0.16 (-0.04-0.35) | 1.10 (0.86-1.33) | 1.07 (0.99-1.15) | <0.0001 |
| NO_2_ | 0.09 (-0.27-0.46) | 1.71 (1.21-2.21) | 1.13 (1.04-1.21) | <0.0001 |  | 0.09 (-0.16-0.33) | 0.72 (0.41-1.03) | 0.76 (0.70-0.82) | <0.0001 |
| O_3_ | 0.61 (0.22-1.00) | 4.42 (3.91-4.92) | 2.00 (1.82-2.11) | <0.0001 |  | 0.34 (0.07-0.60) | 1.55 (1.23-1.87) | 1.28 (1.19-1.38) | <0.0001 |

CI indicates confidence interval; NO_2_, nitrogen dioxide; O_3_, ozone; PM_10_, particle with aerodynamic diameter ≤10 µm; and SO_2_, sulfur dioxide.

^a^Adjusted by age, sex, race, education, income, smoking, drinking, exercise, diet, sugar intake, family history of hypertension, GDP, and PD.

^b^Estimate was scaled to the interquartile range (IQR) for each pollutant (19 µg/m^3^ for PM_10_, 20 µg/m^3^ for SO_2_, 9 µg/m^3^ for NO_2_, and 22 µg/m^3^ for O_3_).

**Table S7** Associations between air pollutants and blood pressures in two BMI categories after excluding participants with diabetes mellitus (n = 15,829)

|  | Systolic blood pressure | | |  |  | | Diastolic blood pressure | |  |
| --- | --- | --- | --- | --- | --- | --- | --- | --- | --- |
|  | Normal weight | Overweight | Pollutant*overweight |  |  | Normal weight | Overweight | Pollutant*overweight |  |
| Pollutant | Estimate (95% CI)^ab^ | Estimate (95% CI)^ab^ | Estimate (95% CI)^ab^ | *P*-value |  | Estimate (95% CI)^ab^ | Estimate (95% CI)^ab^ | Estimate (95% CI)^ab^ | *P*-value |
| Total |  |  |  |  |  |  |  |  |  |
| PM_10_ | 0.53 (0.22-0.84) | 3.98 (3.57-4.39) | 0.72 (0.67-0.78) | <0.0001 |  | 0.24 (0.03-0.45) | 1.43 (1.17-1.68) | 0.51 (0.48-0.55) | <0.0001 |
| SO_2_ | 0.46 (0.16-0.76) | 3.16 (2.77-3.56) | 1.72 (1.59-1.84) | <0.0001 |  | 0.20 (-0.01-0.40) | 1.18 (0.94-1.43) | 1.18 (1.10-1.27) | <0.0001 |
| NO_2_ | 0.10 (-0.28-0.48) | 1.80 (1.27-2.33) | 1.18 (1.09-1.27) | <0.0001 |  | 0.14 (-0.12-0.39) | 0.83 (0.51-1.16) | 0.84 (0.78-0.90) | <0.0001 |
| O_3_ | 0.67 (0.27-1.07) | 4.60 (4.08-5.12) | 2.05 (1.90-2.21) | <0.0001 |  | 0.38 (0.11-0.66) | 1.67 (1.35-2.00) | 1.41 (1.31-1.52) | <0.0001 |

CI, indicates confidence interval; NO_2_, nitrogen dioxide; O_3_, ozone; PM_10_, particle with aerodynamic diameter ≤10 µm; and SO_2_, sulfur dioxide.

^a^Adjusted by age, sex, race, education, income, smoking, drinking, exercise, diet, sugar intake, family history of hypertension, GDP, and PD.

^b^Estimate was scaled to the interquartile range (IQR) for each pollutant (19 µg/m^3^ for PM_10_, 20 µg/m^3^ for SO_2_, 9 µg/m^3^ for NO_2_, and 22 µg/m^3^ for O_3_).
